# A schema for coding health equity scholarship within pediatric research

**DOI:** 10.1017/cts.2024.594

**Published:** 2024-10-02

**Authors:** Najma Abdi, Sabrina W. Tso, Cheyenne Roduin, Elisabeth Nylander, Amanda L. Jones, Susan L. Groshong, Julia Paulsen, Brian E. Saelens

**Affiliations:** 1 Seattle Children’s Research Institute, Seattle, WA, USA; 2 University of Washington Department of Pediatrics, Seattle, WA, USA

**Keywords:** Health equity, health disparity, pediatric research, coding schema (guidelines assessment tools), diversity, equity, and inclusion, scholarship, scholarship/research

## Abstract

**Introduction:**

Seattle Children’s Research Institute is identifying the amount and type of health equity scholarship being conducted institution wide. However, methods for categorizing how scholarship is equity-focused are lacking. We developed and evaluated the reliability of a health equity scholarship coding schema applied to Seattle Children’s affiliated scholarship.

**Methods:**

A 2021–2022 Ovid MEDLINE affiliation search yielded 3551 affiliated scholarship records, with 1079 records identified via an existing filter as scholarship addressing social determinants of health. Through reliability testing and examining concordance and discordance across three independent coders of these records, we developed a coding schema to classify health equity scholarship (yes/no). When health equity scholarship proved positive/Yes, the coders assigned a one through five maturity rating of the scholarship towards addressing inequities. Subsequent reliability testing including a new coder was conducted for 992 subsequent affiliated scholarship records (Oct 2022–June 2023), with additional testing of the sensitivity and specificity of the existing filter relative to the new coding schema.

**Results:**

Reliability for identifying health equity scholarship was consistently high (Fleiss kappas ≥ .78) and categorization of health equity scholarship into maturity levels was moderate (Fleiss kappas ≥ .47). The coding schema identified additional health equity scholarship not captured in an existing filter for social determinants of health scholarship. Based on the new schema, 23.3% of Seattle Childrens’ affiliated scholarship published October 2002–June 2023 was health equity focused.

**Conclusions:**

This new coding schema can be used to identify and categorize health equity scholarship to help quantitate the health equity focus of portfolios of human-focused research.

## Introduction

Health inequities exist in the U.S. as evidenced by differences, often driven by systemic racism and oppression, in health care and outcomes based on characteristics such as gender, race, ethnicity, socioeconomic status, language, and geography [1]. Health equity is the “principle underlying a commitment to reduce—and, ultimately, eliminate—disparities in health and in its determinants, including social determinants [2].” Pursuing health equity means striving for the highest possible “standard of health for all people and giving special attention to the needs of those at greatest risk of poor health, based on social conditions [2].” Inequities exist for nearly all health conditions and health care access and quality between disenfranchized and non-disenfranchized populations [2]. In 2021, racism was considered a public health crisis, given the inequities highlighted through the COVID-19 pandemic and the social unrest of police violence against the Black community. This has led to a paradigm shift in increasing the focus of research that is rooted in anti-racism and health equity [3]. This recent research has the potential to inform ways to eliminate health disparities, however, health equity and related issues continue to be understudied [3].

History has shown that medical research often mistreats and causes mistrust amongst diverse populations, leading to the inability to engage such populations in pediatric and other clinical research [4]. Concerns related to engaging populations that are considered disenfranchized are due to their intentional exclusion that often happens in research [5]. A narrative review discussed the characteristics that allow for the exclusion of hard-to-reach populations including (1) individual, or innate factors such as certain disabilities either mentally or physically, (2) structural factors (i.e socioeconomic position, race, or age that affects their involvement in research) [5]. Additionally, experiences with systems such as having experience in incarceration might cause underrepresentation in engagement in research [5]. However, with increased attention to sociodemographic and economic inequities, the need to conduct, support, and promote health equity research is a priority [4]. Recent examples of this include identifying and highlighting the health equity scholarship conducted within a specific field. For instance, the *Journal of Clinical Sleep Medicine* recently curated a collection of articles published in 2021 focused on equity in sleep health. The goal of this was to encourage and bring awareness to research and researchers focused on disparities associated with sleep and acknowledge the inequities of diagnosing and treating sleep disorders among disenfranchized versus enfranchised patients [6]. There has also been a recent proliferation of recommendations for the conduct of health equity research. For example, Castillo *et al*. developed recommendations for how to conduct health research using the Health Equity Research Impact Assessment, which better addresses health inequities and evaluates medical research’s impact on health equity [7]. These recommendations include critical reflection prompts and questions to guide researchers to focus their studies on health equity and highlight the need for conversations about anti-racism and health equity-based to institutions [7].

Research and other academic collectives (e.g., research institutes, schools of public health, schools of medicine) are becoming more interested in conducting and supporting equity scholarship [8]. However, there have been limited attempts to systematically determine whether a project, study, or other piece of scientific scholarship is or is not focused on health equity. Over the decades, health equity research has progressed significantly, marked by a generational framework [8] that traces the maturation of the field. Initially, “first generation” health equity scholarship primarily focused on identifying and documenting disparities between disenfranchized and privileged groups. As the field progressed, subsequent generations delved deeper into the underlying causes of these disparities, with “second generation” research exploring systemic and structural factors contributing to health inequities [8]. “Third generation” scholarship has shifted towards the development of interventions designed to mitigate these disparities, and more recently, “fourth” and higher generations have begun evaluating the effectiveness of these interventions and their scalability [8]. This enhancement reflects a growing sophistication in addressing health equity, moving from descriptive studies to those that are more focused on strategies to improve the health and well-being of disenfranchized populations and ideally centering these populations in strategy development, implementation, and evaluation. For example, the United States Department of Health & Human Services developed a health equity timeline [9] elevating the development of policies and practices from the civil rights era to modern times, emphasizing how historical decisions and legislative changes have influenced the health inequities among different communities in the past 40 years [9]. Recognizing the need for a structured approach to track this progression within our own initiatives, we have adopted a generational framework to categorize and enhance the rigour of health equity scholarship conducted at Seattle Children’s Research Institute. Driven by our elevated commitment to equity and anti-racism, we were motivated to understand the amount and type of health equity scholarship we are conducting across our institution. This could inform ways to increase the amount, quality, and impact of such scholarship to address existing pediatric health inequities.

The Center for Diversity and Health Equity at Seattle Children’s embarked on creating a reliable and efficient coding schema to identify and categorize health equity scholarship. Herein, we describe the development and testing of the schema, the results of applying the schema, and best practices for using the schema in the future, hoping to increase the amount and quality of health equity scholarship.

## Methods

### Identification of affiliated scholarship

The first step was identifying recent scholarship being conducted by investigators affiliated with Seattle Children’s. Seattle Children’s librarians (CR, EN, JP, and SG) used the following steps to obtain a set of affiliated bibliographic records:

- An Ovid Medline search was created to capture Seattle Children’s affiliates using the institution names Seattle Children’s, Children’s Hospital and Regional Medical Center (prior name of Seattle Children’s), and University of Washington, Department of Pediatrics (the academic unit in which many Seattle Children’s investigators are faculty).
- The search was limited by the publication years of 2006 - Current (See Supplementary Materials 1 for full documentation of the search strategies).
- Results from the searches were manually scanned to identify and remove duplicates and records that were falsely identified (e.g., not scholarship by affiliate authors but shared the same name as an affiliate).
- The two most recent complete years (2021–2022) of records were selected resulting in 3551 bibliographic records.
- This set of records were passed through an existing filter seeking to identify scholarship focused on the social determinants of health developed by Prady *et al*. [11] The filter was modified to include more recent medical subject headings (MeSH). This resulted in 1079 records.
- The final 1079 records were uploaded into EndNote and smart groups were created to sort the publications by year.

### Development of the coding schema

We (NA, SWT, BES) developed an initial set of definitions and instructions to code scholarship as health equity scholarship (yes/no), and if yes, what stage/generation of health equity scholarship is being conducted. The initial coding schema was created from our experiences in conducting health equity research, having roles at promoting and facilitating health equity-focused research, and the 2014 Health Equity Report from the Association of American Medical Colleges that examined the health equity focus among a grant portfolio of 23,000 health services projects [10]. The coding schema was modified based on feedback from colleagues and other local investigators who have expertise in health equity scholarship, including members of the Integrated Special Populations team as part of the Institute for Translational Health Sciences, our regional NIH Clinical Translational Science Award program at University of Washington, Fred Hutchinson Cancer Research Center, and Seattle Children’s.

Starting with the 1079 records from 2021 to 2022 that were identified by the equity search filter, three coders (NA, ST, BES) independently coded the first 150 abstracts from the scholarship or full articles from each record when abstracts were not available or were unclear as to whether health equity was a focus within the scholarship. Coders then convened to examine concordance and discordance, and refinements were made to the coding schema (e.g., more details and examples provided to better differentiate between generations of health equity scholarship). This process was repeated with subsequent subsets of abstracts consisting of four test sets (records 151–300, 301–500, 501–700, 701–1079). Prior to coders convening to examine concordance/discordance for these test sets, reliability across all three coders and by coder pairs was examined for identification of health equity scholarship (yes/no) and generation coding for health equity scholarship. Additionally, coders evaluated 1000 randomly selected records (out of 2472 records) from 2021 to 2022 that were not identified in the social determinants of health search filter to examine whether the search filter successfully captured health equity scholarship and to help refine the coding schema.

### Coding schema

The coding schema is applicable to scholarship focused on human(s), including research with people (e.g., human subjects research), research examining health conditions of people even if people were not directly involved (e.g., examining specimens from individuals with a health condition), or scholarship directly relevant to people or their health care (e.g., reviews or commentaries about the health or care for people). The scholarship record did not have to focus solely on health equity issues to be considered health equity scholarship (e.g., if one analysis among many explored sex differences, then the scholarship record would be coded as health equity scholarship). Scholarship could be quantitative or qualitative, inquiry-based (generating new knowledge), summary (e.g., review), or be a clinical or research commentary, editorial, or guidelines/recommendations. Coders were encouraged to read the title and abstract of each record at least twice, as well as review the full article if necessary to determine whether and how it was health equity scholarship. The full coding schema is provided in supplemental materials 2, under Health Equity Coding Scheme

### Defining health equity scholarship

Briefly, health equity scholarship was defined as scholarship that examines differences between disenfranchized and non-disenfranchized individuals or populations, intentionally focuses on or includes mostly disenfranchized individuals/populations or those living or receiving care in an underserved context or seeks to develop or examine strategies to improve the health or well-being of disenfranchized individuals/populations. We define disenfranchized populations as those who face systemic oppression (e.g., racism) tied to their cultural and personal identities. For example, these include individuals who identify as: Black, Indigenous, Hispanic, or as other people of color (BIPOC), speaking a language other than English, as LGBTQ + or gender diverse, as female, or are lower income or publicly insured or un- or under-insured. Health equity scholarship was also considered scholarship conducted in or with individuals/populations in contexts that are underserved, defined as settings or locations with limited to no access to resources due to geographical isolation (e.g., rural areas), educational disenfranchisement, and/or otherwise resource limited settings (e.g., low-and-middle income country). Other categories defined as health equity scholarship included scholarship: (1) utilizing strategies to increase equitable access for diverse populations to engage in research and (2) examining impacts of racism, sexism, xenophobia, or other oppression with the intention to reduce harm or improve care among marginalized populations. Scholarship that did not meet any of these criteria was not considered health equity scholarship.

Some examples of scholarship that included identity or context information but were not considered health equity scholarship included: (1) reporting information on identity or demographics of participants (e.g., percentage of participants by race/sex) but does not examine differences or the experiences of participants by identity or context; (2) examines a disease or health condition that has inequities in prevalence or negative health outcomes (most do) but does not explore inequities by identity or context within that health condition; (3) makes reference to equity, inequity, or disparities in the background or conclusion but does not examine or deeply address these issues in the methods or results; and (4) includes languages other than English in other countries if that language is the native language of the research participants (e.g., surveying of providers in Spain in Spanish language).

Among records considered health equity scholarship, each was coded into the generation of the scholarship, with earlier generations of scholarship focused on identifying differences or disparities. Later generations focused on examining interventions or other strategies for improving health outcomes and/or health care access and quality for disenfranchized populations or those in underserved contexts.

#### First generation

Scholarship that explored a difference in health behaviours, health condition/outcome, health care, or quality or other factors related to health between those with versus without a disenfranchized identity/context; these are generally descriptive or observational and non-interventional studies and include guidelines/recommendations/commentaries about the general importance of examining equity, racism, or other forms or aspects of oppression.

#### Second generation

Scholarship that sought to obtain a better understanding of the experience of people with a disenfranchized identity or within an underserved context to examine the nature or mechanism of inequity. Participants in second generation research are predominantly individuals with a disenfranchized identity or in an underserved context.

#### Third generation

Scholarship that explored the development, acceptability, or feasibility of an intervention among or for people with disenfranchized identities or within an underserved context or one that serves people who are disenfranchized.

#### Fourth generation

Scholarship that studied the effects or the impact of an intervention on the health, health behaviors, health care access or quality that is specific or tailored to or mostly effects individuals/populations with disenfranchized identities or within underserved contexts, including interventions that address the reason or mechanism of disenfranchisement or seeks to directly impact an inequity.

#### Fifth generation

Exploration of effects or impacts on health, health behaviors, or health care access or quality of an intervention among those with intersecting disenfranchized identities or between disenfranchized identities and underserved context.

### Further testing of the coding schema

To further examine reliability and explore the sensitivity and specificity of the Prady *et al*. [11] filter relative to the coding schema, scholarship records that were affiliated with Seattle Children’s were identified for October 2022–June 2023. Three coders (NA, BES, CR) independently coded the 992 affiliated records, with raters blinded to which records were filtered in or out of the Prady *et al*. [11] filter.

### Analysis

**Figure 1.**
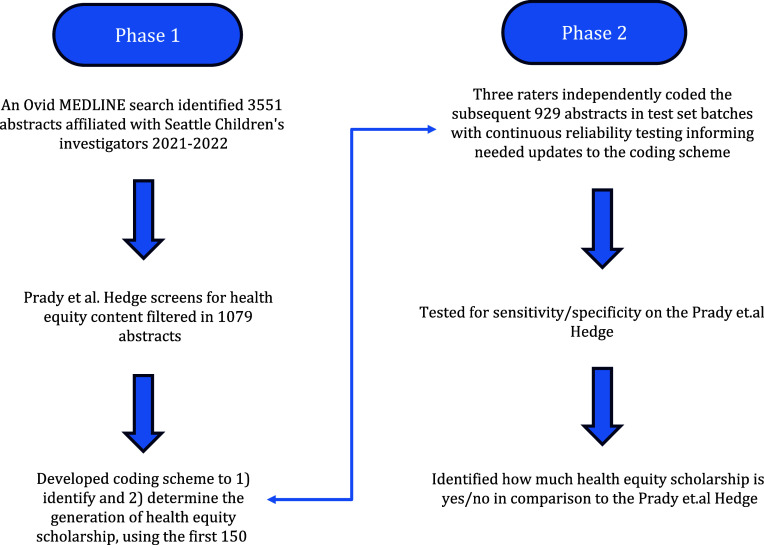
Identifying scholarship and coding process.

Reliability was assessed by Fleiss kappa and tested the overall agreement across three coders, separately for the identification of health equity scholarship (yes/no), and among records coded as health equity scholarship the generation of health equity scholarship. In addition, Fleiss’ kappa and percent agreement were calculated between each coder and the other coders separately (coding pairs). Reliability was assessed separately for the four test sets in the initial coding schema refinement and then among the 992 affiliate records from October 2022–June 2023. After reliability testing, the coders convened to resolve discrepancies and came to consensus for coding on each record. Sensitivity and specificity of the Prady *et al*. [11] and colleagues filter were then calculated for this latter record set using the consensus across coders from the schema as the standard. Figure 1 includes a flow diagram of the coding process.

## Results

Results of the inter-rater reliability testing during the formative phase of the coding schema are presented in Table 1. For the identification of whether records were health equity scholarship or not, overall reliability across the three coders was good/very good (and increased slightly from earlier to later test sets), as was absolute percent agreement and reliability between each pair of coders. For the health equity scholarship generation coding, overall reliability across the three coders was moderate, as was absolute percent agreement, with improvements from earlier to later test sets. The reliability for health equity scholarship generation coding based on each pair of coders ranged from fair to very good, with improvements seen in later test sets.

**Table 1.** Inter-rater reliability for health equity scholarship within each test set among the 2021–2022 Seattle Children’s affiliated scholarship records using the Prady *et al*. [11] filter

	Identification of Health Equity Scholarship (yes/no)			Categorizing Generation (first–fifth) among Health Equity Scholarship		
	Percent agreement between pairs	Fleiss’ kappa		Percent agreement between pairs	Fleiss’ kappa	
Test Sets		Across 3 coders	Range for pair coders		Across 3 coders	Range for pair coders
1^st^ test set: 150 records	88%–94%	0.79	0.75–0.86	57%–73%	0.47	0.30–0.60
2^nd^ test set: 200 records	85%–95%	0.80	0.71–0.90	60%–84%	0.50	0.36–0.71
3^rd^ test set: 200 records	86%–99%	0.81	0.72–0.98	62%–93%	0.55	0.37–0.88
4^th^ test set: 379 records	88%–98%	0.83	0.75–0.96	72%–93%	0.67	0.54–0.87

Inter-rater reliability results for the coding of the subsequent scholarship are presented in Table 2. Even with the addition of a novice coder, overall reliability across the three coders and pairwise reliability including percent agreement were good/very good for the identification of health equity scholarship. The reliability for generation coding was moderate overall and varied for the coder pairs from fair to very good.

**Table 2.** Inter-rater reliability for health equity scholarship coding of Seattle Children’s affiliated scholarship October 2022–June 2023

Identification of Health Equity Scholarship (yes/no)			Categorizing Generation (1^st^–5^th^) among Health Equity Scholarship		
Percent agreement between pairs	Fleiss’ kappa		Percent agreement between pairs	Fleiss’ kappa	
	Across 3 coders	Range for pair coders		Across 3 coders	Range for pair coders
89%–99%	0.78	0.68–0.96	54%–92%	0.48	0.26–0.87

For the 2021–2022 scholarship, the coding schema categorized fewer abstracts as health equity scholarship than the Prady *et al*. [11] filter. However, among the 1000 randomly selected records for coding, only 72 (7.2%) were filtered out of the Prady *et al*. [11] filter but were still coded as health equity scholarship based on our schema. The subsequent comparison on the October 2022–June 2023 scholarship involved blinding the coders to whether scholarship was identified in the Prady *et al*. [11] filter. These results are detailed in Table 3. The Prady *et al*. [11] filter has sensitivity of 58.4% and a specificity of 92.3% relative to the coding schema for the identification of health equity scholarship.

**Table 3.** Identification of health equity scholarship by the Prady *et al*. [11] compared to the coding schema

		Health equity scholarship based on the coding schema		
		No	Yes	Total
Identified in the Prady *et al*. [11] filter	No	706	96	802
	Yes	55	135	190
		761	232	992

**Figure 2.**
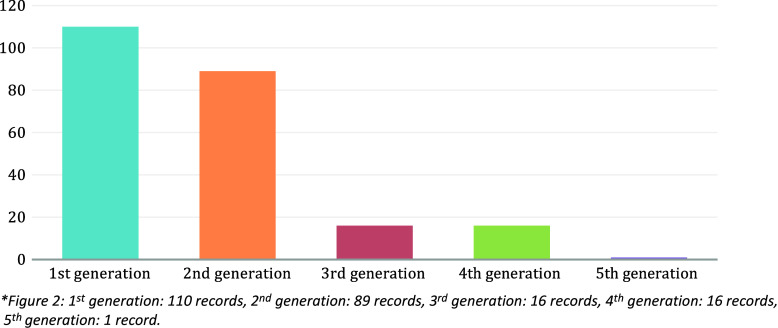
Number of records in each generation coded as health equity scholarship*. * first generation: 110 records, second generation: 89 records, third generation: 16 records, fourth generation: 16 records, fifth generation: 1 record.

Based on the reconciliation of coding for the Seattle Children’s scholarship records from October 2022–June 2023, 23.3% (232/992) of the affiliated scholarship records were identified as health equity scholarship involving humans. In addition, the category of scholarship not considered health equity also included 107 records that were not focused on humans (i.e, research using mouse models). When excluding these records, the percentage of health equity scholarship changes to 26.2% (232/885). Figure 2 illustrates the generation categories for these 232 records of health equity scholarship during this time period, with the majority being in the first or second generation and fewer in the later generations.

### Limitations and strengths

A significant limitation of our study is the reliance on a complex but potentially incomplete definition of health equity, which may not universally capture or reflect the multifaceted nature of this field or differences on how others define health equity. We acknowledge there are numerous alternative interpretations of the criteria we used and that our study represents an early attempt in a quickly expanding field. As such, it is important for readers to recognize that different definitions could lead to different conclusions about which scholarship is health equity focused or not. Furthermore, it is essential to distinguish between mere differences among groups, which are descriptive, and disparities that are normatively significant and the result of systemic discrimination and disenfranchisement, indicating inequities that require action [12]. The term “health equity” is often used interchangeably with “health equality,” yet these concepts differ critically [12]. Health equality focuses on providing the same resources or opportunities to all, irrespective of their needs, while health equity emphasizes adjustments based on specific group needs to achieve equal outcomes [13]. Given the preliminary nature of this research, more consideration on the nuances between “differences,” “disparities,” and “equity” is necessary. This would enrich the understanding that not all differences are disparities, and not all disparities are inequities [13]. The exploration of these distinctions is crucial for developing more effective and targeted health equity interventions in the future. By explicitly problematizing these concepts, we can better understand the limitations of our methodology and refine our approaches to more accurately assess and address health inequities.

We developed and evaluated the reliability of the coding schema focusing on paediatric medicine and health in a single institution within the United States. The practice of health equity research may differ across discipline and across institutions, which might contribute to the limitations of the present coding schema. Additionally, there are likely differences in how people or institutions would define health equity scholarship, especially in the global context and this coding scheme should be adjusted accordingly. For example, there is more science documenting health disparities between disenfranchized and non-disenfranchized populations (i.e., defined herein as first generation) compared to science focused interventions to improve health outcomes among disenfranchized populations (i.e., defined herein as third generation or higher). Some might argue that disparities research examines whether things are equal rather than equitable and thus does little to improve health equity, and thus should not be considered health equity scholarship. The use of the generational coding allows for users to shift the line on which generation they consider the start of health equity scholarship. Recognizing that health disparities exist is just the beginning. Ideally more research would be focused on understanding the determinants associated with those disparities and creating and testing interventions to improve the health outcomes of disenfranchized populations.

Work on the coding schema is in the early stages. Future work could include more validation of the proposed coding schema (e.g., through academic and community expert deliberation and consensus processes). The coding schema was developed and applied to research conducted with/on people or those who investigated specimens from people. It was not clear how to code more basic science (e.g., research at the cellular or sub-cellular level) scholarship for its health equity content so it was deemed not health equity scholarship in the present coding; however, this is an opportunity for future exploration. Furthermore, the coding schema is generally able to be applied based on the scholarship record abstract, although instances exist where it is encouraged to review the entire piece of scholarship (e.g., research article, guidelines/recommendations, tables) to accurately code. This adds to the amount of time it takes for coding. There are other aspects of equitable research not captured in the coding system. For example, the engagement and centering of community is critical to the equitable practice of research and occurs along a continuum which is a critical component of addressing health equity. Community involvement is crucial for understanding the unique needs and experiences of different populations. However, the present coding system does not evaluate whether or how community engagement is included in the scholarship or other ways that the research is being done equitably.

## Discussion

### Public health implications

Researchers and institutions can use the developed coding schema to systematically identify and categorize health equity scholarship. This could lead to a better understanding of and cataloguing of research focused on disenfranchized populations and contexts. Categorization helps raise awareness and prioritize health equity in research. Institutions can use this awareness to allocate resources, funding, track EDI research and publishing, and provide support for projects that aim to reduce health disparities and move towards creating interventions that are categorized as higher generation health equity scholarship. Further, use of the schema could assist the evaluation of strategic research initiatives to improve the quantity and type (e.g., moving from less to more latter generation work) of health equity scholarship, along with other metrics of the strength of health equity research within an institution. For example, Yousefi Nooraie *et al*. [14] used network analysis tools to understand the various equity-related activities of individuals within an academic medical center, including who was perceived to have expertise in racial and ethnic equity research. The coding schema and other tools could be used to examine the impact of large investments in diversifying the research workforce, enhancing community engagement across an institution, and even state laws focused on increasing the racial and ethnic diversity of research participation (Washington State House Bill 1745; passed April 19, 2023).

The present coding schema introduces ways to categorize research based on generation, allowing for the evaluation of impact/interventions and strategies on health outcomes. This can inform future research directions and policy decisions. Furthermore, given the dynamic nature of health equity research, resources should be made available to support the ongoing development of best practices and frameworks for identifying relevant and meaningful health equity research. Additionally, we value the importance of foundational frameworks that have guided the development of our coding schema. Kilbourn *et al*. [15] provides an essential conceptual framework that underscores the progression of health disparities research. Kilbourne *et al*. [15] argue that advancing health disparities research requires both the production of latter generations of research and the continuation of early generational approaches. They emphasize that early generational research, which focuses on detecting and understanding disparities, is fundamental for the ongoing development and sustainability of health equity interventions.

We acknowledge that the schema is specific to this time period and the U.S. research context (although included the evaluation of studies conducted in other countries) and anticipate the coding schema will change substantially due to this continued development of health equity scholarship and hopefully with elimination or at least reduction in disenfranchisement.

### Best practices for future researchers

Our health equity scholarship coding schema can be used to reliably identify health equity scholarship as defined herein. The high reliability results for this identification suggest that a single trained coder can identify health equity scholarship. The variability in reliability for the generation coding suggests the need for multiple raters and consensus convening to ensure accurate coding of health equity scholarship generation.

Both the unblinded and blinded investigation of the present coding schema relative to the filter by Prady *et al*. [11] suggests the latter tends to filter out most scholarship that would not be considered health equity focused, but it may be over-identifying some scholarship relative to the developed coding schema. If it is not feasible to code a large set of scholarship records via the present coding schema, the Prady *et al*. [11] filter may be a good initial filter to apply to reduce the size of the set for subsequent coding of health equity scholarship generation, although the sensitivity of this filter relative to the schema is modest.

By incorporating foundational frameworks, we highlight the need for a multi-generational approach in health equity research. Insights found by Thomas *et al*. [8] and Kilbourne *et al*. [15] inform our understanding that while latter generation research (third, fourth, and fifth generation) is critical for developing and evaluating interventions, early-generation research (first and second generation) remains vital for identifying and understanding the root causes of health inequities. This balanced approach enhances the rigour and impact of health equity scholarship, ensuring that interventions are both innovative and grounded in a thorough understanding of existing inequities. An additional consideration should be ensuring that findings from health equity research inform policy making and advocacy efforts, thereby extending the impact of research beyond academic circles into real-world applications.

## Conclusion

This manuscript addresses the critical need for systematic approaches to identify, categorize, and promote health equity scholarship. By addressing gaps in understanding and prioritizing health equity, institutions can contribute to the broader goal of reducing and eliminating health disparities among diverse populations. The methodology presented in the paper provides a valuable approach towards evaluating and identifying metrics for institutions seeking to enhance their commitment to health equity research.

## Supporting information

Abdi et al. supplementary material 1 Abdi et al. supplementary material

Abdi et al. supplementary material 2 Abdi et al. supplementary material

## Supplementary material

The supplementary material for this article can be found at https://doi.org/10.1017/cts.2024.594.
