# Supplementary material for: A schema for coding health equity scholarship within pediatric research
Source: J Clin Transl Sci. 2024 Oct 2;8(1):e141. doi: 10.1017/cts.2024.594 (PMC11523018; doi:10.1017/cts.2024.594)
Supplement: Abdi et al. supplementary material 1 — Abdi et al. supplementary material [file S2059866124005946sup001.docx]

**Affiliation Search Strategies**

Ovid MEDLINE(R) and Epub Ahead of Print, In-Process, In-Data-Review & Other Non-Indexed Citations, Daily and Versions <1946 to November 02, 2022>

1. (seattle children* or seattlechildren* or "children's hospital & regional medical center" or "children* hospital & regional medical center" or "children's hospital and regional medical center" or "children* hospital and regional medical center" or ("department of pediatrics" adj5 "university of washington")).ia,in.[investigator affiliation, institution] 15089

2. limit 1 to yr="2006 -Current" 13531

**Affiliation & Health Equity Search**

The health equity search is based on the following search filter:

Prady, S. L., Uphoff, E. P., Power, M., & Golder, S. (2018). Development and validation of a search filter to identify equity-focused studies: reducing the number needed to screen. *BMC Medical Research Methodology*, *18*(1), 1-9.

The following modifications to MeSH were made:

- exp Ethnic Groups/ updated to Ethnicity - https://www.ncbi.nlm.nih.gov/mesh/68005006
- exp Continental Population Groups/ updated to Racial Groups - https://www.ncbi.nlm.nih.gov/mesh/68044469

Ovid MEDLINE(R) and Epub Ahead of Print, In-Process, In-Data-Review & Other Non-Indexed Citations, Daily and Versions <1946 to November 02, 2022>

1. (seattle children* or seattlechildren* or "children's hospital & regional medical center" or "children* hospital & regional medical center" or "children's hospital and regional medical center" or "children* hospital and regional medical center" or ("department of pediatrics" adj5 "university of washington")).ia,in. 15089

2. limit 1 to yr="2006 -Current" 13531

3. potential determinants.mp. 2154

4. significant correlates of.mp. 1355

5. (independent correlates or independent association*).mp. 12765

6. variables associated with.mp. 14402

7. determinants of.mp. 186102

8. factors associated with.mp. 162664

9. identif* determinants.mp. 2759

10. (more likely or less likely or just as likely).mp. 344587

11. risk factors for.mp. 1199415

12. (significantly related to or significant predictor).mp. 65055

13. (also adj2 associated with).mp. 76336

14. (at increased risk or at decreased risk).mp. 43004

15. association* between.mp. 656638

16. (positively associated or negatively associated).mp. 85407

17. differed by.mp. 14692

18. (were high* amongst or were low* amongst).mp. 1465

19. (inverse relationship with or inversely associated with or inversely related to).mp. 46888

20. reverse association.mp. 262

21. differentially affects.mp. 2546

22. evidence of a link between.mp. 1910

23. (significantly adj3 likelihood of).mp. 3219

24. protective factors for.mp. 18505

25. (differ* adj2 according to).mp. 33834

26. (inverse adj2 gradient).mp. 274

27. (positive adj2 gradient).mp. 471

28. (negative adj2 gradient).mp. 405

29. (trends were adj3 across).mp. 1926

30. (related to adj3 variable*).mp. 26570

31. (differences were adj3 explained by).mp. 8683

32. (significant among or no# significant among).mp. 4084

33. or/3-32 2464022

34. Residence Characteristics/ 37681

35. Environment design/ 7122

36. exp Marital status/ 39696

37. neighbo?rhood*.mp. 38857

38. residential environment*.mp. 898

39. rural*.mp. 195129

40. inner?city.mp. 56

41. housing instability.mp. 594

42. housing insecurity.mp. 334

43. housing strain.mp. 5

44. housing security.mp. 71

45. mortgage problems.mp. 0

46. foreclosure.mp. 242

47. eviction*.mp. 939

48. housing loss.mp. 24

49. home repossession*.mp. 2

50. home ownership.mp. 533

51. (repossess* adj3 hous*).mp. 8

52. (repossess* adj3 propert*).mp. 1

53. mortgage delinquency.mp. 8

54. mortgage arrears.mp. 1

55. mortgage debt*.mp. 17

56. overcrowding.mp. 3642

57. (living adj1 (outside or inside or near* or adjacent)).mp. 4185

58. (household adj2 size).mp. 1834

59. (marital status or marriage status).mp. 32763

60. (widow* or cohabit* or divorce* or single parent* or live* alone).mp. 28852

61. or/34-60 343420

62. Cultural Deprivation/ 1175

63. Acculturation/ 6936

64. Culture/ 34043

65. Cross-Cultural Comparison/ 27336

66. Cultural Characteristics/ 16847

67. Cultural Diversity/ 12747

68. Language/ 49666

69. "Transients and Migrants"/ 13772

70. exp "Emigrants and Immigrants"/ 15131

71. Minority groups/ 17074

72. Minority health/ 885

73. Prejudice/ 25465

74. Racism/ 5697

75. Xenophobia/ 89

76. Social Discrimination/ 1542

77. exp Race Relations/ 8256

78. exp Ethnicity/ 103852

79. exp Racial Groups/ 243831

80. Refugees/ 12650

81. minorit*.mp. 98700

82. migration background.mp. 800

83. racial.mp. 72273

84. racism.mp. 10371

85. ethnology.mp. 174160

86. race.mp. 134571

87. ethnic*.mp. 211835

88. non?English.mp. 24

89. language other than.mp. 588

90. latino*.mp. 40801

91. latina*.mp. 5226

92. hispanic*.mp. 73524

93. whites.mp. 88280

94. caucasian*.mp. 68265

95. non?white.mp. 3143

96. Torres Strait Islander.mp. 2148

97. aboriginal.mp. 9917

98. native american.mp. 4606

99. inuit.mp. 2026

100. eskimo.mp. 726

101. first nation*.mp. 5981

102. indigenous.mp. 42331

103. english as a second language.mp. 501

104. foreign language.mp. 1612

105. or/62-104 867748

106. Occupations/ 24383

107. Unemployment/ 7682

108. occupations.mp. 48141

109. unemployment.mp. 17101

110. or/106-109 64710

111. exp Gender Identity/ 23028

112. Women's Health/ 29222

113. gender differences.mp. 32290

114. (sex disparit* or sex difference?).mp. 44765

115. gender identity.mp. 23411

116. sex role.mp. 1699

117. wom#n* role?.mp. 688

118. m#n* role?.mp. 11383

119. gender* role?.mp. 3881

120. servicewomen.mp. 132

121. Sex factors/ 278543

122. or/111-121 388991

123. exp Educational status/ 58421

124. Education/ 21504

125. Schooling.mp. 9845

126. educational status.mp. 61212

127. (education* adj2 level?).mp. 62336

128. ((higher or better or worse or less) adj educated).mp. 6542

129. ((higher or better or worse or less) adj level? of education).mp. 2796

130. or/123-129 144394

131. Religion/ 15644

132. religi*.mp. 70330

133. or/131-132 70330

134. Social determinants of Health/ 5879

135. Psychosocial Deprivation/ 2051

136. Sociological Factors/ 715

137. Working Poor/ 18

138. Hierarchy, Social/ 2353

139. disparit*.mp. 115903

140. inequalit*.mp. 46414

141. inequit*.mp. 17766

142. equity.mp. 27708

143. deprivation.mp. 97864

144. gini.mp. 1930

145. concentration index.mp. 2013

146. Socioeconomic Factors/ 170278

147. Social Welfare/ 9686

148. exp Social Class/ 44813

149. exp Poverty/ 48954

150. Income/ 33571

151. Social class*.mp. 50363

152. social determinants.mp. 16228

153. social status.mp. 6932

154. social position.mp. 1172

155. social background.mp. 1269

156. social circumstance*.mp. 1378

157. socio-economic.mp. 38394

158. socioeconomic.mp. 249756

159. sociodemographic.mp. 65823

160. socio-demographic.mp. 31461

161. SES.mp. 25098

162. disadvantaged.mp. 16716

163. impoverished.mp. 4064

164. poverty.mp. 69516

165. economic level.mp. 1315

166. assets index.mp. 24

167. income*.mp. 183633

168. or/134-167 779950

169. Social Stigma/ 12060

170. social capital/ 1531

171. Social Control, Informal/ 3659

172. exp Social Support/ 78495

173. exp Social Environment/ 125308

174. Trust/ 12265

175. Social conditions/ 9563

176. Social isolation/ 15839

177. Social marginalization/ 578

178. Anomie/ 473

179. social participation/ 3288

180. social exclusion.mp. 2428

181. (social adj (capital or cohes* or organis* or organiz*)).mp. 9548

182. (community adj3 (cohes* or participa*)).mp. 40022

183. ((neighbourhood or neighborhood) adj cohes*).mp. 223

184. social relationships.mp. 7342

185. social network*.mp. 25879

186. collective efficacy.mp. 605

187. civil society.mp. 2446

188. informal social control.mp. 136

189. neighbo*rhood disorder.mp. 288

190. social disorgani?ation.mp. 253

191. anomie.mp. 611

192. social support.mp. 103253

193. social participation.mp. 6257

194. trust.mp. 50026

195. emotional support.mp. 7793

196. psychosocial support.mp. 5309

197. community capital.mp. 24

198. neighbo*rhood cohesion.mp. 218

199. social influence.mp. 2637

200. (soci*context* or soci*-context*).mp. 13755

201. or/169-200 325326

202. Health Status Disparities/ 19237

203. Health Services Accessibility/ 84129

204. Health Equity/ 3082

205. health*care disparit*.mp. 22835

206. health care disparit*.mp. 1940

207. health status disparit*.mp. 19620

208. health disparit*.mp. 19132

209. health inequalit*.mp. 7965

210. health inequit*.mp. 4158

211. medically underserved.mp. 8588

212. or/202-211 145195

213. 61 or 105 or 110 or 122 or 130 or 133 or 168 or 201 or 212 2369014

214. 33 or 213 4232577

215. 2 and 214 3929
