# Supplementary material for: A schema for coding health equity scholarship within pediatric research
Source: J Clin Transl Sci. 2024 Oct 2;8(1):e141. doi: 10.1017/cts.2024.594 (PMC11523018; doi:10.1017/cts.2024.594)
Supplement: Abdi et al. supplementary material 2 — Abdi et al. supplementary material [file S2059866124005946sup002.docx]

June 18, 2024

Brian Saelens, Najma Abdi

Seattle Chidlren’s Research institute

Coding scholarship for its health equity focus

Contents

[Introduction 2](#_Toc142557778)

[Coding schema 4](#_Toc142557779)

[Stage/generation of the health equity scholarship 7](#_Toc142557780)

[**1^st^ Generation** 7](#_Toc142557781)

[**2^nd^ Generation** 7](#_Toc142557782)

[**3^rd^ Generation** 8](#_Toc142557783)

[**4^th^ Generation** 9](#_Toc142557784)

[**5^th^ Generation** 9](#_Toc142557785)

[Table 1: Examples of Scholarship that is **NOT c**onsidered health equity 10](#_Toc142557786)

[Examples abstracts that are **NOT** considered health equity. 11](#_Toc142557787)

[Table 2: Examples of health equity scholarship by generation 13](#_Toc142557788)

[Examples of health equity abstract coding with generations: 14](#_Toc142557789)

Coding scholarship for its health equity focus

# Introduction

Purpose | The purpose of this coding is to categorize: 1) type of scholarship, 2) whether or not the scholarship, involving human subjects, is health equity focused 3) the “generation” of the identified health equity scholarship. The generation tracks the direction the scholarship is making towards discovering or implementing solutions to address inequities and/or improve the health and well-being of populations that have been disenfranchised and under-served.

The overall intention of this coding is to efficiently specify the amount/proportion and type of health equity scholarship within a research portfolio (e.g., research division, department, school, institute, etc.).

Scope | This coding is limited to research done with or regarding people (e.g., recruited humans, used data from patients or participants, used biosamples from individuals with a specific health condition who have or could have identities or be within underserved contexts). Also included are reviews or summaries of studies involving humans, or an editorial/commentary/policy statement/clinical guideline or research recommendations specific to humans and/or a health condition or the health care of humans. Note this scope more broadly defines human subject research than others (e.g., federal guidelines). This scope excludes research conducted at more basic biologic levels (e.g., animal research, cellular research that is not specific to a health condition and does not include biosamples from humans, animal-only models of disease) as different definitions for equity scholarship need to be developed for more basic science research.

Source of Material | The source material for coding is published abstracts derived from research databases (e.g., PubMed, Medline) and associated full manuscripts if necessary. The study’s title and any part of the abstract narrative, such as objective, aims, methods, results, and conclusion (although typically found in the methods and/or results) are used to identify whether the scholarship is health equity focused and if so the generation of health equity scholarship. It is sometimes recommended to examine the full article/materials (see guidance on “When to review the full manuscript or article” under section Coding Schema).

Additional notes about coding

- For the purpose of this coding, populations that are disenfranchised are defined as those that have been historically disempowered and oppressed or groups that have limited or no access to resources. Examples of this are those who are socioeconomically disadvantaged, who speak a language other than English, who identify as BIPOC (Black, Indigenous, or other People of Color), who identify as female, who identify as LGBTQ+ or gender diverse, and/or individuals living with a disability.
- Underserved context is defined as geographically underserved areas (e.g., rural area in the U.S.), receiving care or being in less resourced settings (e.g., Federally Qualified Health Centers, juvenile justice centers, Title 1 schools) or in low-and-middle-income countries (also defined as the Global South) that do not have access to resources and/or have a disease/health condition concentrated in a location (i.e., HIV or Malaria in Sub-Saharan Africa)
- Due to the fact that there is lower representation in research participation among those with disenfranchised identities (e.g., race/ethnicity in particular), this coding relies on the assumption that authors/scientists will typically indicate in abstracts that their focus or samples are predominantly from or are regarding disenfranchised populations or contexts.
- It is important to read through the abstract at least twice as equity, identity, and context terms can be subtle and easy to miss.
- This coding will likely develop over time to capture more evolved health equity scholarship and different people or groups may define health equity scholarship differently. For example, we expect there may be disagreement about whether work that explores disparities (1^st^ generation health equity scholarship defined herein) should be considered equity scholarship. Indeed, we anticipate that different entities may use the generational coding to indicate different starting points for their definition of equity scholarship.
- This coding was developed and initially tested on a library of scholarship from a pediatric health research institution (Seattle Children’s Research Institute). However, we believe this coding can be applied more broadly to people-focused health equity scholarship across the life course. This possibility should be rigorously evaluated.
- In this coding, we define disability based on the CDC’s definition of the International Classification of Functioning, Disability, and Health (ICF) (https://www.cdc.gov/ncbddd/disabilityandhealth/disability.html) The ICF conceptualizes a person's level of functioning as a dynamic interaction between individual’s health conditions, environmental factors, and personal factors. It is a biopsychosocial model of disability, based on an integration of the social and medical models of disability.

# Coding schema

Scholarship Type

Characterize the scholarship into: A) ***investigative*** that seeks to contribute new knowledge (qualitative or quantitative that is collecting and reporting on data that is new information), B) ***summary/review*** that seeks to increase knowledge of an area by looking across prior scholarship in that area (e.g., summarizing information across existing studies to identify the most effective intervention; reviews or meta-analysis), or C) ***editorial/commentary/policy statement/clinical guideline or research recommendations*** that takes a position or makes a recommendation about the care or health of individuals/populations or makes about what or how research should be conducted or reported, or D) ***other*** for scholarship that does not fit in the other categories.

Defining people-focused health equity scholarship (code as *Yes*/*No*)

To code ‘Yes’, the scholarship must focus on or address some aspect of human health or well-being *and* the scholarship must be in one or more of the following categories:

- - Intentionally includes or focuses on disenfranchised individuals/populations in the research; this might include:
    1. A focus only on disenfranchised individuals/populations (e.g., how an intervention impacts outcomes among disenfranchised individuals/populations)
    2. Whether differences in health or health outcomes exist by sociodemographic identity characteristics (e.g., race/ethnicity, income status or some proxy like insurance type, gender identity or those with gender dysphoria, sexual identity, or disability status – see note below about developmental delay or disability)
    3. Exploring mechanisms of or reasons for these differences between disenfranchised and non-disenfranchised groups

Note: Not all identity characteristics are included – for example, age, and parent/caregiver sex/gender are not considered disenfranchised identities in this coding

Note: The study can investigate differences but not actually find differences by identity characteristics and still be considered health equity scholarship (e.g., results indicate there are no sex/gender differences in a health condition or provision of health care)

- - The study of people living in underserved areas or receiving care in contexts that often serve those who have been disenfranchised. Examples include, patients receiving care within a Federally Qualified Health Center, youth in foster care, those living in rural areas, care provided in community hospitals, or global health studies that are focused specifically on lower resource or less developed countries or populations therein.
  - The study of a health condition that is overwhelmingly concentrated within a disenfranchised population (e.g., sickle cell disease or malaria)
  - The study focuses on or includes the input or voice of those with a disability regarding their condition, health, or their experience with health care or examines the experience of the interaction between disability and disenfranchised identity or underserved context; or examines the feasibility or health effects of an intervention seeking to improve the health or health care experience of those with a disability
  - The study specifically explores, examines, or describes the impact of racism, sexism, ableism, xenophobia, or other forms of discrimination or oppression based on identity characteristics; or seeks to examine how to reduce racism, bias/harm or to improve the experience of care among disenfranchised individuals/populations (e.g., curriculum to improve health care provider interactions with diverse youth)
  - The study utilizes research methods/strategies that increase equitable access or engagement in research (or examination thereof) among disenfranchised individuals/populations or among those in an underserved context (e.g., community-based participatory research)
- The study provides qualitative or quantitative examination (or description) of the experiences of disenfranchised individuals/populations (e.g., in health care) or in lower resource settings. Example of this would be including languages other than English in study participation, linguistically and/or culturally translating a measure or tool or recruiting more than a modest percentage of participants to engage through a language other than English.

Note: The study does not have to primarily focus on equity. Equity can be defined as present based on one of the specific analyses or a subset of findings within the study. If there are multiple components of a study described (e.g., abstract includes an examination of baseline differences AND intervention effects), be sure to code the generation based on the highest generation equity aspects.

Characteristics of scholarship that is herein defined as **NOT** being health equity scholarship:

- Includes information about identity or demographics of the sample but is not focused on understanding differences by such characteristics, or the experience of disenfranchised individuals/groups or those within underserved contexts, or how interventions are effective among disenfranchised individuals/groups (e.g., provides percentages of participants from different race/ethnic groups in the sample or seeks to match different groups on demographics like through propensity scoring or other methods; examines differences in identity such as sex between two or more samples but does not test whether identity characteristics differ in health, health outcomes, or health care)
- Only adjusts or controls for identity characteristics in analysis but does not examine or report on test for identify characteristic differences in health, health outcomes, or health care by these characteristics.
- Examines costs (e.g., health care costs), but not how identity characteristics interact with costs.
- Examines a disease or health condition that has inequities (most do, so this would be overly inclusive) but does not explore inequities by identity or location/context within that health condition.
- Includes in the research mostly or all people who identify as a single gender/sex (e.g., among individuals who are pregnant, among primary caregivers who in research predominantly include those identifying as female), but does not examine differences by sex/gender or seek to gain a deeper understanding of inequities associated with gender identity (e.g., examining parental/caregiver gender/sex or provider gender/sex is not by itself health equity scholarship) or the intersection of gender/sex and other disenfranchised identities (e.g., race/ethnicity, income status)
- Education status or other things that are potentially more amenable to change are not disenfranchised factors.
- The equity content is only mentioned or alluded to in the Introduction or Background sections (e.g., highlighting existing inequities that have previously been found) or only in the Conclusion or Discussion section (e.g., such as highlighting how findings may have equity implications or briefly mention the importance of equity with little specificity) (although see note about exploring the full manuscript in this case)
- Author replies or other materials that do not introduce new content around equity.
- Uses languages other than English in other countries if that language is the native language of the research participants (e.g., surveying of providers in Spain in Spanish language), with the exception of lower resource contexts.
- Global or multi-national studies that do not examine experiences or effects specific to lower resource countries or areas; studies outside of the U.S in higher or non-developing country or locations (e.g., Taiwan)
- Examines the experience of those living with a disability but are observational and do not use more participatory research methods to better understand the perspectives and lived experiences of those living with a disability or do not evaluate an intervention that seeks to improve the health or health care of those with a disability.

When to review the full manuscript or article:

- An abstract is unavailable.
- There is a reference in the abstract to examining demographics or other individual or person-level or patient characteristics or geography or context generally, but it is not clear from the abstract whether the characteristics being examined include those focused on equity or whether the study actually examined differences based on identity characteristics or contexts
- There is reference in the abstract of the inclusion of a diverse sample or a community-based sample or active participation by those with disenfranchised identities or within underserved contexts (i.e., conducting surveys or qualitative interviews to understand the lived experience of those with a disability)
- There is a mention of equity/inequity or related issues in the title, conclusion/discussion or other future direction parts of the abstract but it is not otherwise clear whether the content of the scholarship is equity focused
- To further examine results, tables and figures to see whether differences were statistically tested (e.g., included p values, confidence intervals) between those with or without disenfranchised identities (not only that the sample or samples differed in the makeup of those identities)

# Stage/generation of the health equity scholarship

Rate the generation only if identified ‘Yes’ for health equity scholarship. When unsure, code to the higher phase/generation.

**1^st^ Generation**

Exploration or description of an inequity or ***difference*** in health behaviors, health condition/outcome, health care, or other factor related to health between people with versus without disenfranchised identities or in versus outside of an underserved context; 1^st^ generation scholarship is non-interventional

Examples include but are not limited to:

- Exploration of the difference between those with versus without a disenfranchised identity or the intersection of disenfranchised identities (if the difference or association explored is discrimination or related issues, then code as 2^nd^ generation)
- Examining how a health outcome/behavior is associated with disenfranchised identity characteristics (e.g., health condition’s severity varies across different racial/ethnic groups and/or are there gender/sex differences)
- Prevalence of those with a disenfranchised identity within a health condition or context
- Commentaries/editorials that identify or describe differences with at least some detail (generally whole sections or important components of the scholarship focus on this) between disenfranchised and non-disenfranchised groups or indicate that inequities exist (e.g., historical racism) or generally should be addressed and considered, but detailed specific recommendations are not provided (e.g., curated collection of health equity scholarship in a specific field; commentary about the importance of addressing racism)

1^st^ generation terminology might include: modified by race and sex, gender dysphoria differences, income differences, investigated impact of race, income, or sex

### **2^nd^ Generation**

Seeking to obtain a ***better understanding of the experience*** of people with a disenfranchised identity or among those within an underserved context or to examine the nature or mechanism of an inequity or the experience of non-familial providers (professional or non-professional) who care for them. This scholarship has predominantly or is focused all on the participation by/with individuals with a disenfranchised identity or within an underserved context (may need to examine the percentage of people of different racial/ethnic, income, or other identity groups in the study to term predominance).

Examples include but are not limited to:

- - - Surveys, qualitative interviews (not including diagnostic interviews), or biologic or other methods to better understand the experience of mostly or only disenfranchised individuals/populations or those within underserved contexts about health care or for a health condition
    - Surveys or qualitative interviews of providers who care for disenfranchised individuals/populations or within underserved contexts
    - Using languages other than English in surveys or interviews or research focused on linguistic and/or cultural translation of measures or tools
    - Design or protocol descriptions of an intervention focused on disenfranchised populations or addressing inequity not including only those with a disability (e.g., no data or findings about the intervention process or impact)
    - Improving assessment or measurement among disenfranchised individuals/populations or within an underserved context
    - Examining barriers to implementing a clinical guideline, care, or research participation among disenfranchised populations
    - Observational case studies about the non-intervention experience of those with disenfranchised identities or in underserved contexts
    - Examining the experience of the intersection of different disenfranchised identities (e.g., race/ethnic effects by income status)
    - A statement/clinical guideline/recommendation/modeling or framework or summary or review of studies specific to the care for, or treatment of, people with disenfranchised identities or within underserved contexts, or about the equitable practice of research or clinical care
    - Examining differences between those with versus without a disenfranchised identity or in versus outside of underserved contexts, with the focus of the examination of difference being the structure or nature of the marginalization (e.g., examining perceptions of police violence)
    - Examination of an existing intervention or care among those with disenfranchised identities or in an underserved context (either alone or in comparison to those who are not disenfranchised or in an underserved context), but the intervention is not specific, tailored, or modified to/for those disenfranchised identities or contexts.

2^nd^ Generation terminology might include: qualitative study among disenfranchised population, clinical guidelines on health disparities

### **3^rd^ Generation**

Exploration of the development, acceptability, or feasibility ***of an intervention*** (usually new or substantively modified) among and specifically tailored to mostly or only people with disenfranchised identities or within an underserved context (or the intersection of identities and/or location/context).

An intervention can be focused on an individual or group care/treatment, a broad systemic intervention or a policy, or intervention development or simulation, or case study about the care and treatment of those with disenfranchised identities or in an underserved context

Examples include but are not limited to:

- Examining the acceptability, feasibility, or fidelity of an intervention among disenfranchised populations or in an underserved context, without examining the effect or impact on health outcomes, health behaviors, or health care access or quality; this could include conducting a comprehensive assessment of multiple stakeholders (e.g., community-based providers) of what is feasible, appropriate, or what should be considered in the study design or implementation of an intervention specific to a disenfranchised population or in an underserved context
  - - Examining the impact of an intervention on knowledge or attitudes about health or health behaviors among a disenfranchised population or in an underserved context, without measuring the effect of the intervention on health outcomes, health behaviors, or health care access or quality

3^rd^ generation terminology might include: acceptability/feasibility of intervention, community-based, intervention testing

### **4^th^ Generation**

Exploration of the ***effects or the*** ***impact of an intervention*** (usually new or substantively modified) on the health, health behaviors, health care access or health care quality that is specific or tailored to or mostly effects individuals/populations with disenfranchised identities or within underserved contexts. The intervention does not necessarily have to target the source of disenfranchisement – can focus on secondary or other effects (e.g., care for disenfranchised people with an outcome not necessarily the source of the disenfranchisement). This generation also includes interventions that address the reason or mechanism of disenfranchisement or seeks to directly address the source of the inequity.

Examples include but are not limited to:

- - - The effects of an intervention specifically designed for a disenfranchised population on the health and well-being of disenfranchised populations, including those with a disability, or those within disenfranchised populations or underserved contexts
    - The effects of an intervention to change the care that disenfranchised populations or those within disenfranchised populations or underserved contexts receive (e.g., effects of providing professional interpretation during clinical encounters)
    - The effects of a policy on health, health behaviors, or related outcomes of those from disenfranchised populations or in underserved contexts (e.g., earned income tax credit effects on mental health outcomes)

4^th^ generation terminology might include: intervention effects, evaluated health outcomes, impact on health, health model, quality improvement.

### **5^th^ Generation**

Exploration of effects or impacts on the health, health behaviors, health care access or health care quality of an ***intervention among those with an intersection*** of different disenfranchised identities or between disenfranchised identity and underserved context (looking at two or more disenfranchised identity factors separately does not constitute intersectionality)

Examples include but are not limited to:

- The effects of an intervention on lower income members of those with disenfranchised racial/ethnic identities
- The effects of an intervention conducted within a lower resource setting (e.g., FQHC, lower income country) among those with disenfranchised racial/ethnic identities.
- The effects of an intervention among individuals living with a disability and live in an underserved context (rural area or low-income country) or have another disenfranchised identity (race/ethnicity, sex, low-income)

5^th^ generation terminology might include mention and focus of intersectional identities (e.g., examining gender affirming care for transgender youth in Malawi)

# Table 1: Examples of Scholarship that is **NOT c**onsidered health equity scholarship

|  | **Not health equity research** | **The focus of the research is not on people** | **Non-investigative article that is not considered health equity** |
| --- | --- | --- | --- |
| **Article Details** | L.P. Richardson: Electronic Health Risk Behavior Screening with Integrated Feedback Among Adolescents in Primary Care: Randomized Controlled Trial | S.V.J Rasmussen: Functional genomic analysis of epithelioid sarcoma reveals distinct proximal and distal subtype biology | D.J. Opel: Amplifying Appeals to the Common Good in COVID-19 Vaccine Messaging |
| **Coded as** | Not health equity scholarship | The focus of the research is not on people | Not health equity scholarship |
| **Reason** | Study aimed to test efficacy of electronic risk behavior screening in primary care. Did not recruit a diverse population or have a specific focus on health equity. | Article highlighted complexity of metastatic epithelioid sarcoma through EPS cultures. People were not included in the study. | This article falls under being a “commentary”. It is an opinion piece for individuals to get vaccinated. Does not address health equity issues or focus on disenfranchised populations/health outcomes |

# Examples abstracts that are **NOT** considered health equity scholarship

This section provides abstracts for each example above

- Coded as **not being health equity scholarship:**

L.P. Richardson: Electronic Health Risk Behavior Screening with Integrated Feedback Among Adolescents in Primary Care: Randomized Controlled Trial

Health risk behaviors are the most common sources of morbidity among adolescents. Adolescent health guidelines (Guidelines for Preventive Services by the AMA and Bright Futures by the Maternal Child Health Bureau) recommend screening and counseling, but the implementation is inconsistent. This study aims to test the efficacy of electronic risk behavior screening with integrated patient-facing feedback on the delivery of adolescent-reported clinician counseling and risk behaviors over time. This was a randomized controlled trial comparing an electronic tool to usual care in five pediatric clinics in the Pacific Northwest. A total of 300 participants aged 13-18 years who attended a well-care visit between September 30, 2016, and January 12, 2018, were included. Adolescents were randomized after consent by employing a 1:1 balanced age, sex, and clinic stratified schema with 150 adolescents in the intervention group and 150 in the control group. Intervention adolescents received electronic screening with integrated feedback, and the clinicians received a summary report of the results. Control adolescents received usual care. Outcomes, assessed via online survey methods, included adolescent-reported receipt of counseling during the visit (measured a day after the visit) and health risk behavior change (measured at 3 and 6 months after the visit). Of the original 300 participants, 94% (n=282), 94.3% (n=283), and 94.6% (n=284) completed follow-up surveys at 1 day, 3 months, and 6 months, respectively, with similar levels of attrition across study arms. The mean risk behavior score at baseline was 2.86 (SD 2.33) for intervention adolescents and 3.10 (SD 2.52) for control adolescents (score potential range 0-21). After adjusting for age, gender, and random effect of the clinic, intervention adolescents were 36% more likely to report having received counseling for endorsed risk behaviors than control adolescents (adjusted rate ratio 1.36, 95% CI 1.04 to 1.78) 1 day after the well-care visit. Both the intervention and control groups reported decreased risk behaviors at the 3- and 6-month follow-up assessments, with no significant group differences in risk behavior scores at either time point (3-month group difference: β=-.15, 95% CI -0.57 to -0.01, P=.05; 6-month group difference: β=-.12, 95% CI -0.29 to 0.52, P=.57). Although electronic health screening with integrated feedback improves the delivery of counseling by clinicians, the impact on risk behaviors is modest and, in this study, not significantly different from usual care. More research is needed to identify effective strategies to reduce risk in the context of well-care.

- Coded as **the focus of the research is not on people** so not considered heath equity scholarship in this coding:

S.V.J Rasmussen: Functional genomic analysis of epithelioid sarcoma reveals distinct proximal and distal subtype biology

Metastatic epithelioid sarcoma (EPS) remains a largely unmet clinical need in children, adolescents and young adults despite the advent of EZH2 inhibitor tazemetostat. In order to realize consistently effective drug therapies, a functional genomics approach was used to identify key signaling pathway vulnerabilities in a spectrum of EPS patient samples. EPS biopsies/surgical resections and cell lines were studied by next-generation DNA exome and RNA deep sequencing, then EPS cell cultures were tested against a panel of chemical probes to discover signaling pathway targets with the most significant contributions to EPS tumor cell maintenance. Other biologically inspired functional interrogations of EPS cultures using gene knockdown or chemical probes demonstrated only limited to modest efficacy in vitro. However, our molecular studies uncovered distinguishing features (including retained dysfunctional SMARCB1 expression and elevated GLI3, FYN and CXCL12 expression) of distal, paediatric/young adult-associated EPS versus proximal, adult-associated EPS. Overall results highlight the complexity of the disease and a limited chemical space for therapeutic advancement. However, subtle differences between the two EPS subtypes highlight the biological disparities between younger and older EPS patients and emphasize the need to approach the two subtypes as molecularly and clinically distinct diseases.

- **Non-investigative article** that is coded as not being health equity research:

D.J. Opel: Amplifying Appeals to the Common Good in COVID-19 Vaccine Messaging

“We are all in this together.” This appeal to solidarity and the common good from the World Health Organization’s One World: #TogetherAtHome campaign at the start of the COVID-19 pandemic exemplified the promise of a silver lining: for all its pain and suffering, our sense of obligation to one another could get us through. Appeals like this have purpose during times of fear and uncertainty. Elimination of an infectious disease, given its transmissibility, demands it. Today, this message feels distant and idealistic, and the spirit of solidarity it conveys has remained maddeningly elusive. Even the availability of a vaccine, typically a means in which to appeal to the common good, has been mired in a debate about individual risk and benefit. For instance, rather than creating an ethos of kinship, vaccine requirements in workplaces, restaurants, concert venues, and universities have instead fueled disagreements about the nature and magnitude of disease risk and whether this risk justifies infringement on individual liberties. In this Viewpoint, we highlight how appeals to self-interest as a means to encourage vaccination have come to prevail in the pandemic. We argue for the need to transition from this approach to appeals that reclaim a spirit of solidarity. We suggest 3 strategies to promote COVID-19 vaccination anchored in our obligations to one another.

# Table 2: Examples of health equity scholarship by generation

|  | **1^st^ generation** | **2^nd^ generation** | **3^rd^ generation** | **4^th^ generation** | **5^th^ generation** | **Non-investigative health equity scholarship examples** |
| --- | --- | --- | --- | --- | --- | --- |
| **Article Details** | E.L Neuhaus: Resting State EEG in Youth with ASD: age, sex, and relation to Phenotype | S.M.U Simkovich: Resources and Geographic Access to Care for Severe Pediatric Pneumonia in Four Resource-limited Settings | G.M.K Sequeira: Increasing the frequency of Affirmed Name and Pronoun Documentation in Pediatric Emergency Department | M.I.K. Patel: Association of a Lay Health Worker-Led Intervention on Goals of Care, Quality of Life, and Clinical Trial Participation Among Low-Income and Minority Adults with Cancer | R.L.S Drieling: Randomized trial of a portable HEPA air cleaner intervention to reduce asthma morbidity among Latino children in an agricultural community | D. Yeboah: Language Matters: Why We Should Reconsider the Term Limited English Proficiency (1^st^ generation)  A. Kamath: Tailoring the perioperative surgical home for refugee families (2^nd^ generation) |
| **Coded as** | Yes, health equity scholarship.  1^st^ generation | Yes, health equity scholarship.  2^nd^ generation | Yes, health equity scholarship.  3^rd^ generation | Yes, health equity scholarship.  4^th^ generation | Yes, health equity scholarship.  5^th^ generation | Yes, health equity scholarship 1^st^ and 2^nd^ generations respectively |
| **Reason** | Article examined sex differences among youth with Autism Spectrum Disorder. | Article conducted a comprehensive survey to understand pediatric pneumonia in low-and-middle-income countries. | Article used a quality improvement approach to seek to increase frequency of pronoun/name documentation in the emergency department (care changed) | Article conducted a lay health worker–led intervention(s) that resulted in improved supportive care, improved quality of life, and clinical trial participation among low-income and minority populations with cancer (care changed and improved outcomes) | Article described the results of using a HEPA air cleaner intervention on health outcomes among Latino youth living in agricultural/rural communities. | 1^st^ generation: This article falls under being a “commentary/ editorial note” as it highlights the issue of categorizing families as “limited-English proficient” and is calling for a change in terminology that is equitable and inclusive.  2^nd^ generation: This article falls under being a “summary/ review” and presents conceptual frameworks on refugee policies and specific recommendations on how to equitably address the perioperative healthcare of refugee children. |

# Examples of health equity abstract coding with generations:

This section provides abstracts for each example above. The highlighted parts of the abstract indicate how health equity scholarship was identified and how the generation of health equity scholarship was determined.

- **Coded as ‘Yes’ health equity scholarship, 1^st^ generation:**

E.L Neuhaus: Resting State EEG in Youth with ASD: age, sex, and relation to Phenotype

Identification of ASD biomarkers is a key priority for understanding etiology, facilitating early diagnosis, monitoring developmental trajectories, and targeting treatment efforts. Efforts have included exploration of resting state encephalography (EEG), which has a variety of relevant neurodevelopmental correlates and can be collected with minimal burden. However, EEG biomarkers may not be equally valid across the autism spectrum, as ASD is strikingly heterogeneous and individual differences may moderate EEG-behavior associations. Biological sex is a particularly important potential moderator, as females with ASD appear to differ from males with ASD in important ways that may influence biomarker accuracy. We examined effects of biological sex, age, and ASD diagnosis on resting state EEG among a large, sex-balanced sample of youth with (N = 142, 43% female) and without (N = 138, 49% female) ASD collected across four research sites. Absolute power was extracted across five frequency bands and nine brain regions, and effects of sex, age, and diagnosis were analyzed using mixed-effects linear regression models. Exploratory partial correlations were computed to examine EEG-behavior associations in ASD, with emphasis on possible sex differences in associations. Decreased EEG power across multiple frequencies was associated with female sex and older age. Youth with ASD displayed decreased alpha power relative to peers without ASD, suggesting increased neural activation during rest. Associations between EEG and behavior varied by sex. Whereas power across various frequencies correlated with social skills, nonverbal IQ, and repetitive behavior for males with ASD, no such associations were observed for females with ASD. Research using EEG as a possible ASD biomarker must consider individual differences among participants, as these features influence baseline EEG measures and moderate associations between EEG and important behavioral outcomes. Failure to consider factors such as biological sex in such research risks defining biomarkers that misrepresent females with ASD, hindering understanding of the neurobiology, development, and intervention response of this important population.

- **Coded as ‘Yes’ health equity scholarship; 2^nd^ generation article:**

S.M.U Simkovich: Resources and Geographic Access to Care for Severe Pediatric Pneumonia in Four Resource-limited Settings

Pneumonia is the leading cause of death in children worldwide. Identifying and appropriately managing severe pneumonia in a timely manner improves outcomes. Little is known about the readiness of healthcare facilities to manage severe pediatric pneumonia in low-resource settings. As part of the HAPIN (Household Air Pollution Intervention Network) trial, we sought to identify healthcare facilities that were adequately resourced to manage severe pediatric pneumonia in Jalapa, Guatemala (J-GUA); Puno, Peru (P-PER); Kayonza, Rwanda (K-RWA); and Tamil Nadu, India (T-IND). We conducted a facility-based survey of available infrastructure, staff, equipment, and medical consumables. Facilities were georeferenced, and a road network analysis was performed. Of the 350 healthcare facilities surveyed, 13% had adequate resources to manage severe pneumonia, 37% had pulse oximeters, and 44% had supplemental oxygen. Mean (±SD) travel time to an adequately resourced facility was 41 ± 19 minutes in J-GUA, 99 ± 64 minutes in P-PER, 40 ± 19 minutes in K-RWA, and 31 ± 19 minutes in T-IND. Expanding pulse oximetry coverage to all facilities reduced travel time by 44% in J-GUA, 29% in P-PER, 29% in K-RWA, and 11% in T-IND (all P < 0.001). Most healthcare facilities in low-resource settings of the HAPIN study area were inadequately resourced to care for severe pediatric pneumonia. Early identification of cases and timely referral is paramount. The provision of pulse oximeters to all health facilities may be an effective approach to identify cases earlier and refer them for care and in a timely manner.

- **Coded as ‘Yes’ health equity scholarship; 3^rd^ generation:**

G.M.K Sequeira: Increasing the frequency of Affirmed Name and Pronoun Documentation in Pediatric Emergency Department

In a previous study of 204 transgender and gender diverse youth in our region, 44% reported being made to feel uncomfortable in the emergency department (ED) because of their gender identity. The objective of our study was to conduct a 2 year quality improvement project to increase affirmed name and pronoun documentation in the pediatric ED. Using process mapping, we identified 5 key drivers and change ideas. The key driver diagram was updated as interventions were implemented over 3 Plan-Do-Study-Act cycles. Our primary outcome, the percentage of ED visits per month with pronouns documented, was plotted on a run chart with the goal of seeing a 50% increase in form completion from a baseline median of ∼14% over the 2 year study period. The frequency of pronoun documentation increased from a baseline median of 13.8% to a median of 47.8%. The most significant increase in pronoun documentation occurred in Plan-Do-Study-Act cycle 3, immediately after ED-wide dissemination of a near-miss case and subsequent call for improvement by ED leadership. Roughly 1.7% of all encounters during the study period involved patients whose pronouns were discordant from the sex listed in their electronic health record. This quality-improvement project increased the frequency of pronoun documentation in the ED. This has the potential to improve the quality of care provided to transgender and gender diverse youth in the ED setting and identify patients who may benefit from receiving a referral to a pediatric gender clinic for additional support.

- **Coded as ‘Yes’ health equity scholarship; 4^th^ generation:**

M.I.K. Patel: Association of a Lay Health Worker-Led Intervention on Goals of Care, Quality of Life, and Clinical Trial Participation Among Low-Income and Minority Adults With Cancer

New approaches are needed to overcome low supportive care and clinical trial participation among low-income and minority adults with cancer. The objective of this project was to determine whether a lay health worker intervention was associated with improvements in supportive care and trial participation. We conducted a quality improvement initiative in collaboration with a union organization. We enrolled union members newly diagnosed with cancer into a 6-month lay health worker-led intervention from October 15, 2016, to February 28, 2017. The primary outcome was goals of care. Secondary outcomes were health-related quality of life (HRQOL), health care use, and trial participation. All outcomes except HRQOL were compared with a cohort of union members diagnosed within the 6-month preintervention period. Sixty-six adults participated in the intervention group, and we identified 72 adults in the control group. Demographic characteristics were similar between groups. The mean age was 56.0 years; 47 (34%) were male, and 22 were White (16%). Within 6 months enrollment, more intervention group participants, as compared with the control, had clinician-documented goals of care (94% *v* 26%; *P* < .001) and participated in cancer clinical trials (72% *v* 22%; *P* < .001). At 4 months postenrollment, as compared with baseline, intervention participants experienced HRQOL improvements (mean difference, 3.98 points; standard deviation, 2.83; *P* < .001). Before death, more intervention group participants used palliative care and hospice than the control group. Lay health worker-led interventions may improve supportive care and clinical trial participation among low-income and minority populations with cancer.

- **Coded as ‘Yes’ health equity scholarship; 5^th^ generation article:**

R.L.S Drieling: Randomized trial of a portable HEPA air cleaner intervention to reduce asthma morbidity among Latino children in an agricultural community

Data on pediatric asthma morbidity and effective environmental interventions in U.S. agricultural settings are few. We evaluated the effectiveness of HEPA air cleaners on asthma morbidity among a cohort of rural Latino children. Seventy-five children with poorly controlled asthma and living in non-smoking homes were randomly assigned to asthma education alone or along with HEPA air cleaners placed in their sleeping area and home living room. The Asthma Control Test (ACT) score, asthma symptoms in prior 2 weeks, unplanned clinical utilization, creatinine-adjusted urinary leukotriene E4 (uLTE4 [ng/mg]), and additional secondary outcomes were evaluated at baseline, six, and 12 months. Group differences were assessed using multivariable-adjusted generalized estimating equations. Incident rate ratios of ever experiencing the metrics of poorer asthma health during follow-up (suboptimal asthma management) were estimated using Poisson regression models in secondary analysis. Mean child age was 9.2 and 8.6 years in intervention and control groups, respectively, and two-thirds of participants were male. Primary analysis of repeated measures of ACT score did not differ between groups (HEPA group mean change compared to controls 10% [95% CI: - 12-39%]). A suggestion of greater decrease in uLTE4 (ng/mg creatinine) was observed (- 10% [95% CI: - 20 -1%]). Secondary analysis showed children with HEPAs were less likely to have an ACT score meeting a clinically defined cutoff for poorly controlled asthma using repeated measures (IRR: 0.45 [95% CI: 0.21-0.97]). In Poisson models, intervention participants had reduced risk of ever meeting this cutoff (IRR: 0.43 [95% CI: 0.21-0.89]), ever having symptoms in the past 2 weeks (IRR: 0.71 [95% CI: 0.52-0.98]), and lower risk of any unplanned clinical utilization (IRR: 0.35 [95% CI: 0.13-0.94]) compared to control participants. The HAPI study showed generally improved outcomes among children in the HEPA air cleaner group. However, primary analyses did not meet statistical significance and many outcomes were subjective (self-report) in this unblinded study, so findings must be interpreted cautiously. HEPA air cleaners may provide additional benefit for child asthma health where traditional asthmagens (traffic, tobacco smoke) are not prominent factors, but larger studies with more statistical power and blinded designs are needed.

- **Coded as ‘Yes’ health equity scholarship and non-investigative health equity article:**

**1^st^ generation**

Yeboah: Language Matters: Why We Should Reconsider the Term Limited English Proficiency

Health care systems in the United States continue to face challenges in optimizing medical care for a diverse and growing multilingual patient population. Research has demonstrated numerous health disparities in the medical care provided to patients who use a non-English language for medical communication. Differences in care have led to more serious adverse events, longer hospital stays, and lower patient satisfaction for individuals who use a non-English language for medical care. A commitment to providing equitable care for non–English-speaking patients requires removing the label “limited English proficiency” (LEP) that has been widely used in clinical care and research for decades. It is time to examine the negative connotations embedded within this terminology and propose new terminology that reflects a respectful and responsive approach to providing high-quality care, regardless of patients’ language needs. In an article published in the Journal of Immigrant and Minority Health by Ortega et al, the authors explain key concepts about the problematic assumptions of the LEP terminology. The language surrounding the term LEP reinforces an ethnocentric view of English as the ideal language. Language can be used as an instrument to demonstrate our relative positions of power or lack thereof in a particular setting. When patients who use a non-English language are labeled as LEP, it centers on English and reinforces its importance as the dominant language within the health care setting. This notion inadvertently implies that speaking English is central to good communication and thus crucial to quality services and appropriate care. This dominant language ideology contributes to further marginalization of patients who use a non-English language for medical care.

**2^nd^ generation**

Kamath: Tailoring the perioperative surgical home for refugee families.

According to recent United Nations High Commissioner for Refugees (UNHCR) estimates, ~90 million people are forcibly displaced due to conflict, persecution, violence, human rights violations, public disorder, natural disasters, or famine. Of those forcibly displaced, 27.1 million are refugees.^1^ By the 1951 Refugee Convention, a refugee is defined as a person, who, “owing to a well-founded fear of persecution for reasons of race, religion, nationality, membership of a particular social group or political opinion, is outside the country of his nationality and is unable or, owing to such fear, is unwilling to avail himself of the protection of that country.” More than half of all refugees are children. Refugees worldwide have steadily increased, almost tripling over the past decade. Most refugees today, roughly 80%, are hosted by low- and middle-income countries, while the United States receives the largest applications of refugees worldwide. Regardless of a host country’s health system capacity, refugees universally face barriers to access and delivery of quality health services. Children are considered among the most vulnerable throughout the migration process, with regard to health risks, physical and mental well-being, and adverse outcomes. Surgical conditions comprise a large and rising portion of the global burden of disease, but the majority of people around the world, including refugee children, cannot receive safe surgical and anesthesia care when needed. For forcibly displaced persons, there is an estimated surgical need of 3 million procedures annually. At least 60% of refugees live in urban settings, with resettlement in host communities. The area of focus for this work is based on an urban tertiary care center and partnering primary care clinic in the United States. First, we present the conceptual frameworks with regard to refugee policies and perioperative care. Next, we examine the health considerations of refugee children and a case scenario to illustrate this. Finally, we propose an integrated, patient-centered care model to equitably address the perioperative health care of refugee children.

**Examples of when tests of difference in identity characteristics are or are not health equity scholarship**

The part of Table 1 below (from Brown et al. 2023 *Critical Care Medicine*) is reporting of tests for differences (as suggested by the p value) in sex and race differences between the two samples (teens, young adults) that are being examined in this study. It is NOT evidence of health equity scholarship between sex and race differences are not being explored for health, health outcomes, or health care differences based on these identity characteristics.

In contrast, in the part of Table 5 below (also from Brown et al. 2023 *Critical Care Medicine*), the analysis examines and reports out the racial/ethnic differences in survival (a health outcome) based on this identity characteristic, making this health equity scholarship (2^nd^ generation because it explores the existing intervention of life support and racial/ethnic differences of survival from this intervention).
